# A common pathogenic founder variant in Rwandan breast cancer cases

**DOI:** 10.1371/journal.pgen.1012180

**Published:** 2026-11-02

**Authors:** Achille V. C. Manirakiza, Shakuntala Baichoo, Annette Uwineza, Damas Dukundane, Eulade Rugengamanzi, Janviere Mutamuliza, Allison M. O. Niragira, Raissa Muvunyi, Jeffrey Besada, Sarah M. Nielsen, Brianna Bucknor, Diane R. Koeller, Caroline Andrews, Leon Mutesa, Temidayo Fadelu, Timothy R. Rebbeck

**Affiliations:** 1 Oncology Service, Department of Medicine, King Faisal Hospital Rwanda, Kigali, Rwanda; 2 Africa Health Sciences University, Kigali, Rwanda; 3 Center for Human Genetics and Genomics, School of Medicine and Pharmacy, College of Medicine and Health Sciences, University of Rwanda, Kigali, Rwanda; 4 PMCC-AI, University Health Network, Toronto, Canada; 5 Dana Farber Cancer Institute, Boston, Massachusetts, United States of America; 6 Oncology department, Butaro Level II Teaching Hospital, Butaro, Rwanda; 7 Medical Department, University of Global Health Equity (UGHE), Butaro, Rwanda; 8 Research Directorate, Rwanda Military Teaching Hospital, Kigali, Rwanda; 9 Biomedical Services, National Reference Laboratory, Rwanda Biomedical Center, Kigali, Rwanda; 10 Exact Sciences Corp., Madison, Wisconsin, United States of America; 11 Labcorp (formerly Invitae Corp.), San Francisco, California, United States of America; 12 Division of Cancer Genetics and Prevention, Dana-Farber Cancer Institute, Boston, United States of America; 13 Division of Medical Oncology, Dana Farber Cancer Institute, Boston, Massachusetts, United States of America; 14 Harvard Medical School, Boston, Massachusetts, United States of America; 15 Division of Population Science, Dana Farber Cancer Institute, Boston, Massachusetts, United States of America; 16 Department of Epidemiology, Harvard T.H. Chan School of Public Health, Boston, Massachusetts, United States of America; Newcastle University, UNITED KINGDOM OF GREAT BRITAIN AND NORTHERN IRELAND

## Abstract

Germline data from African populations remain sparse, limiting characterization of population-specific *BRCA1*/2 pathogenic variants (PV). In a study of 175 Rwandan women with breast cancer, 7 unrelated carriers (4% of cases; 22% of PV carriers) harbored the same *BRCA1* frameshift PVs, c.4065_4068del (p.Asn1355Lysfs*10), which is extremely rare in gnomAD yet recurrent in European, Asian, and Middle Eastern cohorts. Whole-exome sequencing and haplotype analysis of all 7 carriers revealed a shared ancestral block of approximately 581 kb surrounding the variant, and extended haplotype homozygosity and network analyses confirmed a common founder origin. Coalescent-based age estimation placed the founder event approximately 4,000--10,000 years ago. Comparison with 1000 Genomes Project data showed the founder haplotype is absent or exceedingly rare outside African and South Asian populations. These findings strongly suggest the c.4065_4068del variant as a pre-historical *BRCA1* founder variant in Rwanda, with implications for targeted genetic testing, cascade screening, and cancer prevention in the region.

## Author summary

Breast cancer is rising rapidly across sub-Saharan Africa, yet little is known about inherited gene variants that drive hereditary risk in African women. We investigated a recurring mutation in the *BRCA1* gene, whose normal function protects against breast and ovarian cancer. We observed this mutation at unexpectedly high frequency among Rwandan women with breast cancer. We found that 7 of 175 women tested (4%) carried the exact same *BRCA1* mutation, called c.4065_4068del, despite none being known relatives. To determine whether this recurrence reflected a shared ancestor, we analyzed the DNA surrounding the mutation in all 7 women. All 7 carried a nearly identical DNA stretch around the mutation, a hallmark of what geneticists call a “founder effect,” where a mutation present in one common ancestor spreads to many descendants over generations. Formal relatedness testing confirmed the women are not closely related, meaning this shared mutation traces back thousands of years to a common prehistoric ancestor. All 7 carriers had triple-negative breast cancer, one of the most aggressive subtypes, and most presented with advanced disease. These findings have direct implications for genetic counseling, population-level testing, and cancer prevention in Rwanda and neighboring East African countries.

## Introduction

Breast cancer incidence is rising in sub‑Saharan Africa, with an estimated 200,000 cases diagnosed annually. This increasing burden is accompanied by a disproportionate prevalence of aggressive, early‑onset cases, and triple‑negative breast cancer (TNBC), one of the most aggressive subtypes. Despite this, hereditary variation, particularly germline genomic data from African populations remain sparse [1–3]. *BRCA1* and BRCA2 pathogenic variants (PVs) account for a substantial fraction of hereditary and early‑onset breast cancer globally, but most of what is known about variant spectra, founder effects, and population‑specific risks comes from European and Asian ancestry cohorts [4–9]. Recent work from East and Central Africa [10–12], including Rwanda [13,14], has begun to close this gap by applying multigene cancer susceptibility panels, revealing *BRCA1/2* PV prevalences that are at least comparable to, and in some series higher than, those observed in European populations, and showing strong enrichment of BRCA1 PV among women with triple‑negative breast cancer. These emerging data underscore the need to characterize recurrent and potentially founder PVs in African populations, both to refine risk estimates and to inform context‑appropriate genetic testing strategies.

In a recent Rwandan panel‑testing study of 175 women with breast cancer, 32 (18%) carried at least one germline PV in an established cancer susceptibility gene, and 7 of these carriers (22%; 7/175 overall, 4%) harbored the same *BRCA1* frameshift PV, c.4065_4068del (p.Asn1355Lysfs*10) [13]. This four‑base‑pair deletion in exon 10 induces a frameshift at codon 1355 and a premature termination codon 10 amino acids downstream and is predicted to result in nonsense‑mediated decay or a severely truncated *BRCA1* protein, consistent with its classification as pathogenic in ClinVar and OncoKB. The variant is extremely rare in general‑population datasets (reported in 2–3 of ~245,000–250,000 chromosomes in gnomAD) but has been described repeatedly in breast and ovarian cancer families from European [4,15,16], Asian [6,16,17], Middle Eastern [7,18,19], and North African populations [9], and has been implicated as a founder PV in at least one Pakistani series [8]. The unexpectedly high frequency of c.4065_4068del among otherwise unrelated Rwandan cases, all with similar triple‑negative phenotypes in prior work [13], strongly suggests a founder effect in this population. Systematic study of this PV—its frequency, clinical correlates, and underlying haplotype—therefore offers a tractable opportunity to define a potentially important, population‑specific *BRCA1* founder variant in Rwanda, with direct implications for genetic risk assessment, cascade testing, and tailored screening and prevention strategies in the region.

## Methods

### Ethical statement

The seven (7) individuals were consented for additional testing and publication after the primary analysis [1], as allowed by the larger Institutional Review Board approval (996/RNEC/2021 and 456/RNEC/2022). A formal written and signed consent was obtained from all the seven individuals.

### Participants

Collection and assessment of 175 breast cancer cases was described previously [13]. Briefly, female breast cancer (fBC) were included if they were 1) Diagnosed on or before the age of 45 years; 2) Diagnosed under age 51 years with unknown or limited family history; 3) Diagnosed under age 51 years with a second breast cancer diagnosed at any age; 4) Diagnosed under age 51 years with 1 or more close blood relatives with breast, ovarian, pancreatic, or PC at any age, 5) Diagnosed under age 61 years with triple-negative breast cancer, the age cutoff of the triple-negative breast cancer was 61 years, and patients with this histology subtype were recruited based on this age limit); 6) Diagnosed at any age with 1 or more close blood relatives with breast cancer diagnosed under age 51 years or ovarian, pancreatic, or high-risk PC at any age; 7) Diagnosed at any age with 3 or more total diagnoses of breast cancer in patient and/or close relatives. A panel of 84 cancer-predisposition genes was used to conduct clinical-grade genetic testing on all patients. Full-gene sequencing, deletion/duplication analysis, and variant interpretation were performed at Invitae (now part of Labcorp) ®.

### Statistical methods

#### Study samples and variant.

Whole-exome sequencing (WES) data from 7 unrelated Rwandan individuals, all heterozygous for a pathogenic *BRCA1* frameshift variant (chr17:43,091,462 A > T, proxy for CTTGA>C), were analyzed to characterize the founder haplotype surrounding this variant. The joint-called VCF (GRCh38) was used as the starting point for all downstream analyses.

#### Haplotype phasing and tag SNP selection.

All biallelic variants (SNPs and indels) within a 2 Mb window centered on the PV were extracted with no minor allele frequency filter and a genotyping rate ≥ 90%, yielding 1,114 variants including the pathogenic frameshift. The pathogenic variant itself (chr17:43,091,462 CTTGA>C) was retained as the focal marker; no proxy SNP was required. Linkage disequilibrium (LD) pruning (window 50, step 5, r^2^ < 0.2) selected 53 tag SNPs. Statistical phasing was performed using Beagle 5.4 (27Feb25.75f) with the 1000 Genomes Project (1000G) GRCh38 chr17 phased reference panel and a recombination map, with the pathogenic indel retained through a custom pre-processing step. Dense phasing of all 1,114 biallelic variants was also performed for higher-resolution analyses.

#### Shared haplotype block detection.

For each pair of samples (21 pairs from C (7,2)), the longest shared haplotype block around the PV was identified by extending outward from the PV position and allowing up to 2% allele mismatches to accommodate statistical phasing errors inherent to small sample sizes. For each pair, all four haplotype combinations were tested, and the combination yielding the longest shared block was retained. Physical block lengths (kb) were converted to genetic distances (cM) using interpolation of the GRCh38 genetic map.

#### Variant annotation.

All variants within the *BRCA1* ± 1 Mb window were functionally annotated using SnpEff (v5.4a, database GRCh38.99) to predict variant effects (missense, nonsense, frameshift, splice-site, etc.). Clinical significance was overlaid using SnpSift with the NCBI ClinVar VCF database. Annotated variants were summarized into a table with gene, effect, impact tier (HIGH, MODERATE, LOW, MODIFIER), and ClinVar classification.

#### Population haplotype comparison.

Haplotypes from the 1000 Genomes Project phase 3 (2,548 individuals, 5 superpopulations: AFR, AMR, EAS, EUR, SAS) were extracted at all 394 shared-block variant positions overlapping the 1000G chr17 VCF (∼28-fold increase over the previous 14-SNP panel). Additionally, 112 overlapping markers from the MADCaP continental African cohort (1,561 individuals; 896 cases, 665 controls) were used for African-specific frequency estimation. For each reference haplotype, a similarity score (fraction of matching alleles) to the Rwandan founder haplotype was computed.

#### Extended Haplotype Homozygosity (EHH).

EHH decay was computed using the R package rehh on the densely phased Rwandan VCF and on 1000G population subsets (AFR, EUR, EAS). The pathogenic frameshift was retained in the phased VCF and used directly as the focal marker for EHH and bifurcation analyses. For completeness, analyses using the previously reported proxy SNP (chr17:43,091,983, 521 bp from PV; D’ = 0.67, r^2^ = 0.33 with the PV) are shown in S2 Fig; results are qualitatively consistent but quantitatively less precise.

#### Founder age estimation.

Two complementary approaches were used to estimate the age of the founder PV. First, a simple decay estimate was constructed using the 7-way shared haplotype block (the longest contiguous region identical across all 7 PV-carrying haplotypes), the block length was converted from base pairs to cM using genetic map interpolation, and the founder age was estimated as g = 1/(2L), where L is the block length in Morgans. Second, a pairwise estimate was computed. For all 21 sample pairs, the optimal shared block length around the PV was computed (with 2% mismatch tolerance). The mean block length across all pairs was used in the same formula, with 95% confidence intervals from 10,000 bootstrap iterations. A generation time of 28 years was assumed for both methods.

#### MDS analysis.

Our Rwandan and 1000G haplotypes at overlapping variant positions were merged into a single PLINK dataset, and identity-by-state (IBS) distance was computed. Multidimensional scaling (MDS) was performed to project all individuals into a low-dimensional space for ancestry visualisation, with the 7 Rwandan samples overlaid on the 1000G population structure. To formally test pairwise relatedness among the 7 carriers, KING-robust kinship coefficients and IBS0 fractions were computed across 733,829 autosomal SNPs from the whole-exome data for all 21 pairs (C(7,2)), using a kinship threshold of 0.0442 to define third-degree relatedness. A permutation test assessed whether the observed 7-way shared haplotype core exceeds chance: 10,000 random sets of 7 individuals were drawn from 1000G AFR haplotypes (MAF ≥ 0.10 ascertainment), and the distribution of their maximum identical-by-state-compatible core lengths around the PV position was compared with the observed value.

## Results

Seven of 175 Rwandan women with breast cancer (4% of cases; 22% of all PV carriers) harbored the *BRCA1* c.4065_4068del (p.Asn1355Lysfs*10) PV. None were known to be biologically related. All 7 carriers presented with triple-negative breast cancer, with a mean age at diagnosis of 39.4 years. At presentation, 6 (85.7%) had locally advanced disease and 1 (14.3%) had metastatic disease; none presented with localized disease. This uniform TNBC phenotype and predominantly advanced-stage presentation are consistent with the aggressive clinical behavior previously associated with *BRCA1* loss-of-function PVs in this population. Clinical and demographic details are summarized in Table 1.

**Table 1:** Summary of 175 female breast cancer cases with genetic testing.

PGV			c.4065_4068del (p. Asn1355Lysfs*10) (N = 7, 4%)	Other PV Carriers (N = 25, 14.3%)	No PV Found (N = 143, 81.7%)
**Age at Diagnosis** Mean (SD), (Range)			Mean = 39.4 (SD = 8.52) Range: [33–56]	Mean = 39.8 (SD = 9.60) Range: [25–82]	Mean = 39.8 (SD = 6.4) Range: [20–61]
**Stage at Diagnosis**					
Localized			0 (0%)	12 (38%)	45 (31%)
Locally Advanced			6 (18.8%)	10 (31.2%)	85 (59%)
Metastatic			1 (3.0%)	3 (9.0%)	12 (8.4%)
Unclear			0 (0%)	0 (0%)	1 (0.7%)
**Family History in First and Second-Degree Relatives**					
Breast Cancer			7 (22%)	0 (0%)	10 (7.0%)
Prostate Cancer			0 (0%)	1 (3.1%)	0 (0%)
Ovarian Cancer			0 (0%)	0 (0%)	0 (0%)
Other Cancers			0 (0%)	0 (0%)	0 (0%)
Breast & Prostate Cancer			0 (0%)	0 (0%)	0 (0%)
No Breast or Prostate Cancer			0 (0%)	2 (6.3%)	12 (8.4%)
Unknown/Not Reported			0 (0%)	22 (69%)	121 (85%)
Disease Subtype**					
**ER**	**PR**	**HER2**			
–	–	–	7 (21.9%)	9 (28.1%)	30 (21%)
–	–	+	0 (0%)	2 (6.2%)	20 (14%)
–	+	–	0 (0%)	0 (0%)	2 (1.4%)
–	+	+	0 (0%)	0 (0%)	1 (0.7%)
+	–	–	0 (0%)	1 (3.0%)	11 (7.7%)
+	–	+	0 (0%)	1 (3.0%)	5 (3.5%)
+	+	–	0 (0%)	12 (37.5%)	47 (33%)
+	+	+	0 (0%)	1 (3.0%)	27 (19%)

Fig 1 shows pedigrees of the 7 carriers.

**Fig 1:**
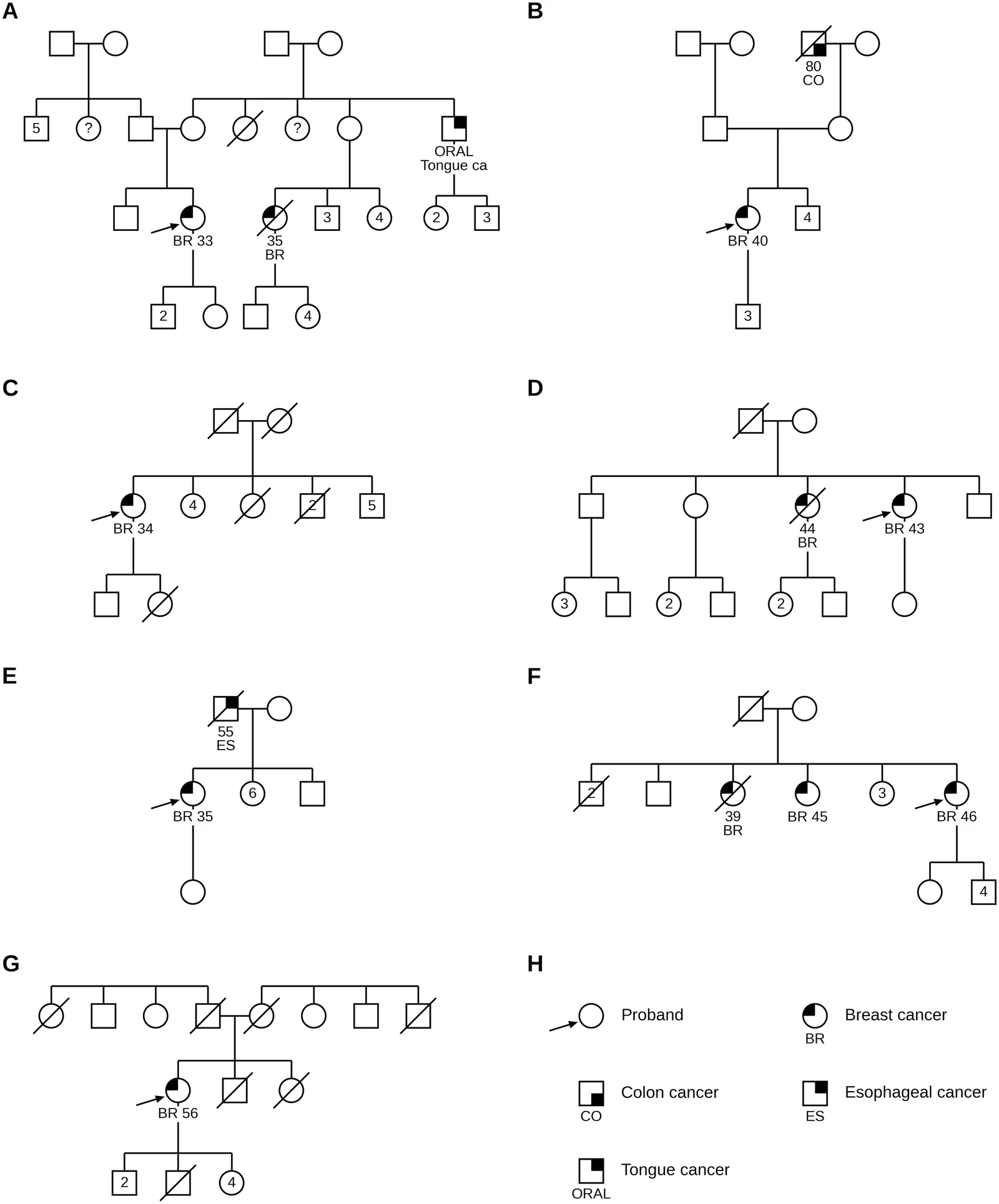
Pedigrees of Putative Founder PV Carriers. A) KS005 Pedigree; B) NMJ0125 Pedigree; C) UP0018 Pedigree; D) MJ07 Pedigree; E) US0127 Pedigree; F) MH0145 Pedigree; G) ME0329 Pedigree; H) Legend.

Table 2 provides detailed clinical information of the patients.

**Table 2:** Detailed disease characteristics of the patients.

Participant	Age at presentation	Stage at presentation	Pathology details	Treatment
KS005	35	III – progressed to IV	Invasive Ductal Carcinoma, NST, Grade 3, Ki-67 score >30%	Chemotherapy, Surgery, Radiation – Palliative chemotherapy (at progression), maintained on Olaparib
MJ007	43	IV	Invasive Ductal Carcinoma, NST, Grade 3, Ki-67 score >50%	Radiation (Brain metastasis) + Chemotherapy (Palliative)
UP0018	43	a. HR + /HER-2: III b. TNBC: IV	a. Invasive Ductal Carcinoma, NST, Grade 2, Ki-67 – 10% b. Invasive Ductal Carcinoma, NST, Grade 3, Ki-67 score 50%	a. HR + /HER2- disease – surgery, chemotherapy, denied radiation therapy, ET b. TNBC (metastatic at presentation) – Palliative chemotherapy, maintained on Olaparib
NMJ0125	40	III	Invasive Ductal Carcinoma with medullary features, grade 3	Breast Conserving Surgery, Chemotherapy, Radiation Therapy
US0127	35	III	Invasive Ductal Carcinoma, NOS, grade 2	Chemotherapy, Breast Conserving Surgery, Radiation Therapy
MH0145	46	III	Invasive Ductal Carcinoma, NST, grade 3	Modified Radical Mastectomy, Chemotherapy, Radiation Therapy
ME0329	56	III	Invasive Ductal Carcinoma, NST, grade 3	Chemotherapy, Breast Conserving Surgery, Radiation Therapy

### Genetic variant analysis

A total of 3,865 variants in the *BRCA1* ± 1 Mb window was functionally annotated. By SnpEff impact tier: 8 HIGH impact (including the pathogenic frameshift), 48 MODERATE (missense variants), 91 LOW (synonymous), and 3,718 MODIFIER (intronic, intergenic). ClinVar annotation identified 398 variants with clinical classifications: 367 Benign, 16 Likely Benign, 11 Benign/Likely Benign, 2 Variants of Uncertain Significance (VUS), 1 Pathogenic (the known *BRCA1* founder variant), and 1 with conflicting classifications. The predominance of benign variants confirms that the shared haplotype block does not harbor additional PVs beyond the known founder variant.

All 7 PV carriers shared a common ancestral haplotype block of approximately 581 kb surrounding the c.4065_4068del variant (chr17:42,650,027–43,231,456), providing strong evidence of a founder effect. Fig 2 shows pairwise linkage disequilibrium (r^2^) between the 48 tag SNPs spanning the *BRCA1* ± 1 Mb window is displayed as a lower-triangle heatmap. The *BRCA1* gene structure is shown in the top annotation track. The shared haplotype block (green shading, 42.65–43.23 Mb) and the PV position (red crosshairs) are marked. A prominent block of high LD (dark red tiles) is visible within the shared region, consistent with the founder haplotype being in strong linkage across this segment.

**Fig 2:**
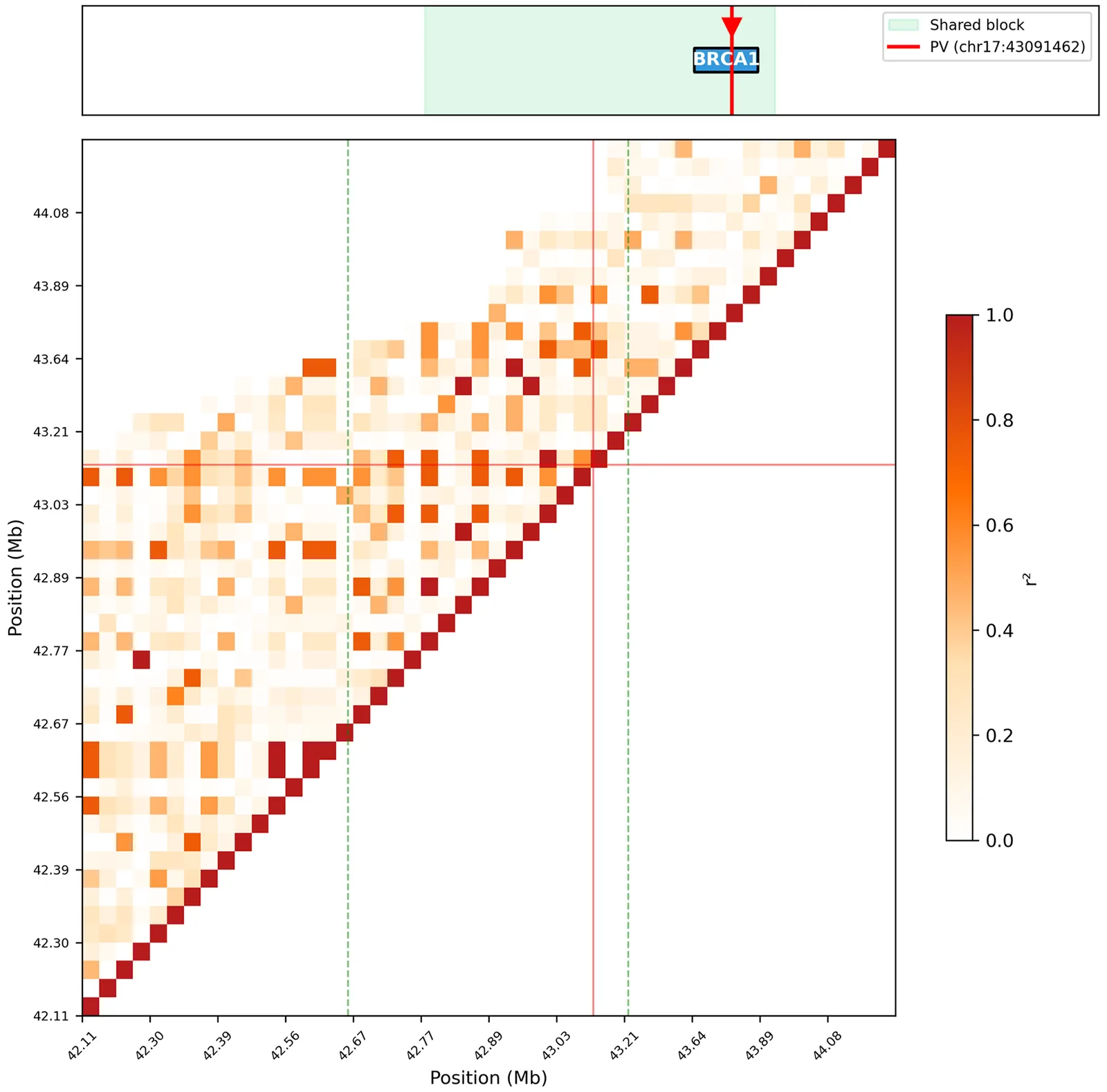
BRCA1 LD structure with gene annotation.

S1 Fig shows the phased haplotype structure across all 14 chromosomes (7 samples × 2 haplotypes). Blue tiles indicate the reference allele, and red tiles indicate the alternate allele at 1,282 informative variant positions across the ± 1 Mb window. The green-shaded region marks the shared haplotype block (~581 kb). The red dashed line indicates the PV at chr17:43,091,462. Within the shared block, a clear pattern of allele identity is visible on the PV-carrying haplotypes, supporting a common founder origin.

Comparison of the Rwandan founder haplotype against 5,096 haplotypes from 1000G populations revealed that African (AFR) and South Asian (SAS) populations showed the highest similarity, with no 1000G haplotype achieving a 100% match; examination of the MADCaP continental African cohort (n = 1,561) confirmed the variant is approximately 60-fold rarer across the broader African population than among Rwandan breast cancer cases. Fig 3 shows haplotype similarity between the Rwandan founder haplotype and 1000G superpopulations; key results are summarized below. Panel A shows violin plots of haplotype similarity scores (fraction of matching alleles at 394 overlapping variant positions) between the Rwandan founder haplotype and 5,096 1000G haplotypes, grouped by superpopulation, with additional comparison against 1,561 MADCaP continental-African individuals. The red dashed line marks the maximum achievable similarity (13/14 = 0.93). African (AFR) populations show the broadest distribution, with 3 haplotypes reaching the maximum similarity. South Asian (SAS) populations show the highest mean similarity (0.489). Panel B shows the frequency of high-similarity haplotypes at three thresholds (>70%, > 80%, > 90%). SAS has the highest proportion at >80% (63 haplotypes, 6.4%), while only AFR and SAS populations contain haplotypes at >90% match. The absence of any 100% match in 1000G is consistent with the founder haplotype being enriched in Rwanda; however, this inference is limited by under-representation of East African populations in 1000G and by the marker density available. Examination of the MADCaP African cohort (n = 1,561) identified a single carrier of c.4065_4068del (carrier frequency 0.06%; allele frequency 3.2 × 10^-4^), confirming the variant is approximately 60-fold rarer in the broader African population than among Rwandan breast cancer cases (4%), consistent with a locally enriched founder allele.

**Fig 3:**
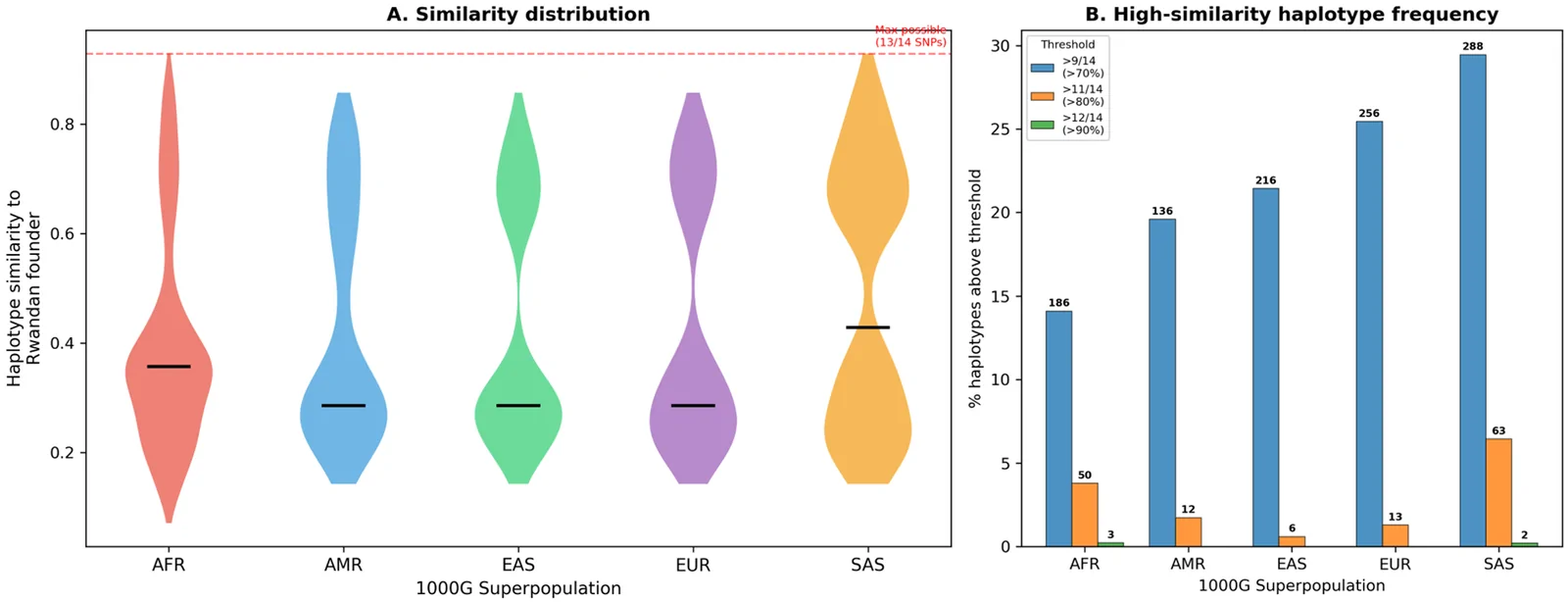
Haplotype similarity between Rwandan founder and 1000 Genomes populations.

EHH analysis revealed extended haplotype homozygosity around the PV in Rwandan carriers, with the ancestral allele reaching peak of 1.0 near the PV and decaying rapidly over ~10 kb, consistent with a founder event in which recombination has not yet fully eroded the shared ancestral segment; the derived allele EHH was near zero throughout. Fig 4a shows EHH decay around the PV in Rwandan carriers. EHH is plotted for the ancestral (red) and derived (blue) alleles at the nearest polymorphic proxy to the PV in the 7 Rwandan samples (14 haplotypes). The ancestral allele shows a sharp peak of haplotype homozygosity near the PV (reaching 1.0) with rapid decay over ~10 kb, consistent with a relatively young founder event that has not yet been broken up by recombination in this small sample. The derived allele EHH is near zero across the region, reflecting very low frequency of the derived allele at this specific proxy position. Note: with only n = 7, EHH estimates are noisy.

**Fig 4:**
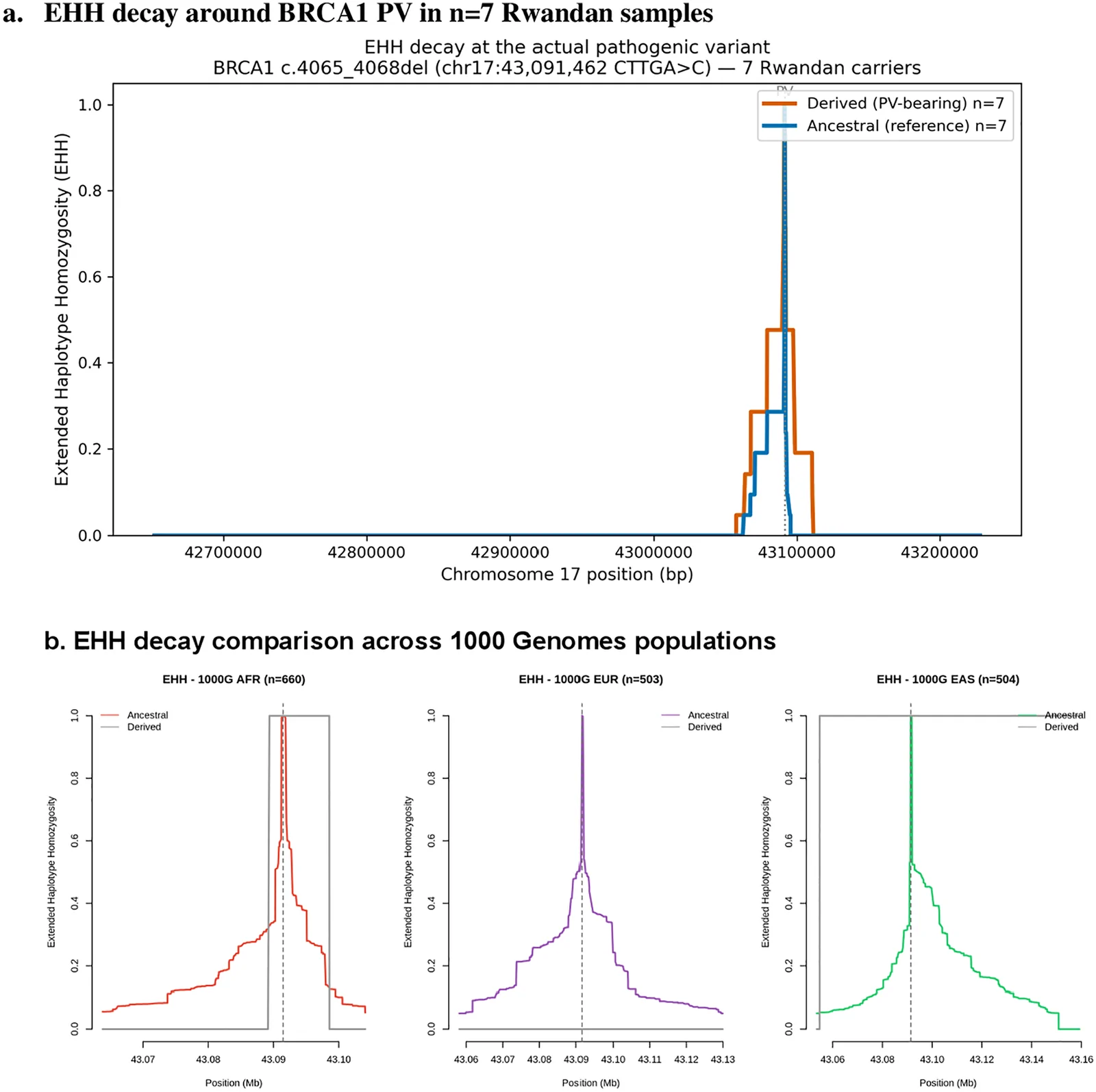
Extended Haplotype Homozygosity (EHH) decay in Rwandan samples. a. EHH decay around BRCA1 PV in n = 7 Rwandan samples. b. EHH decay comparison across 1000 Genomes populations.

For context, EHH decay in three 1000G superpopulations (AFR, EUR, EAS) is shown in Fig 4b for three 1000G superpopulations at the nearest SNP to the *BRCA1* PV: AFR (n = 660, red), EUR (n = 503, purple), and EAS (n = 504, green). In all three populations, the derived allele shows a sharp EHH peak centered on the PV position with rapid decay within ~20 kb. The AFR population shows a broader and more elevated EHH for the ancestral allele, consistent with greater haplotype diversity at this locus in African populations. The sharp EHH peaks in all populations indicate that the variant sits within a region of high haplotype homozygosity, though the pattern does not suggest strong recent positive selection at this locus.

Bifurcation analysis corroborates the EHH findings: PV-associated haplotypes form a single tight cluster near the focal point, while ancestral haplotypes diverge more rapidly. Full bifurcation diagrams are provided in S2 Fig for the ancestral (red, 6 haplotypes) and derived (blue, 8 haplotypes) alleles at a polymorphic proxy SNP (chr17:43,091,983, 521 bp from the PV). Each horizontal line represents one haplotype extending outward from the focal SNP in both directions, with branching points indicating where haplotypes diverge. The derived (blue) haplotypes show tight clustering near the focal point with a single long shared tract, consistent with a recent common ancestor. The ancestral (red) haplotypes diverge more rapidly, indicating greater background haplotype diversity. The dashed line marks the actual PV position (chr17:43,091,462). With only 14 haplotypes, the diagram is sparse but clearly shows the extended sharing among PV-associated haplotypes.

S3 Fig shows pairwise shared block lengths across all 21 carrier pairs, which were strongly right-skewed (median = 0.032 cM, maximum = 0.33 cM), with pairs involving KS005, ME0329, and UP0018 consistently showing the longest blocks (>400 kb), suggesting these three individuals share the most recent common ancestor. Panel A shows block lengths in cM; Panel B shows per-pair block sizes in kb, color-coded by median (green = above, red = below). The mean and median lines are annotated directly below the bars.

The haplotype network confirmed that the three PV-carrying founder haplotypes (KS005 hap2, ME0329 hap2, UP0018 hap1) are virtually identical, differing by only 1–2 SNPs across 14,489 variants in the shared block region — strong evidence of descent from a single common ancestor. Fig 5 shows the haplotype network of 14 phased *BRCA1* haplotypes. Multidimensional scaling (MDS) of pairwise Hamming distances (computed across 14,489 variants in the shared block region) positions the 14 haplotypes in two dimensions. Minimum spanning tree (MST) edges connect haplotypes, with edge labels indicating the number of SNP differences. Each node is coloured by sample. The three PV-carrying founder haplotypes (KS005 hap2, ME0329 hap2, UP0018 hap1; marked [FOUNDER]) cluster tightly on the right with only 1–2 SNP differences, confirming they carry a virtually identical haplotype inherited from a common ancestor. KS005 hap1 and MJ007 hap1 are completely identical (0 differences), forming a shared non-PV haplotype on the left. The remaining haplotypes show 130–530 SNP differences, representing normal background haplotype diversity.

**Fig 5:**
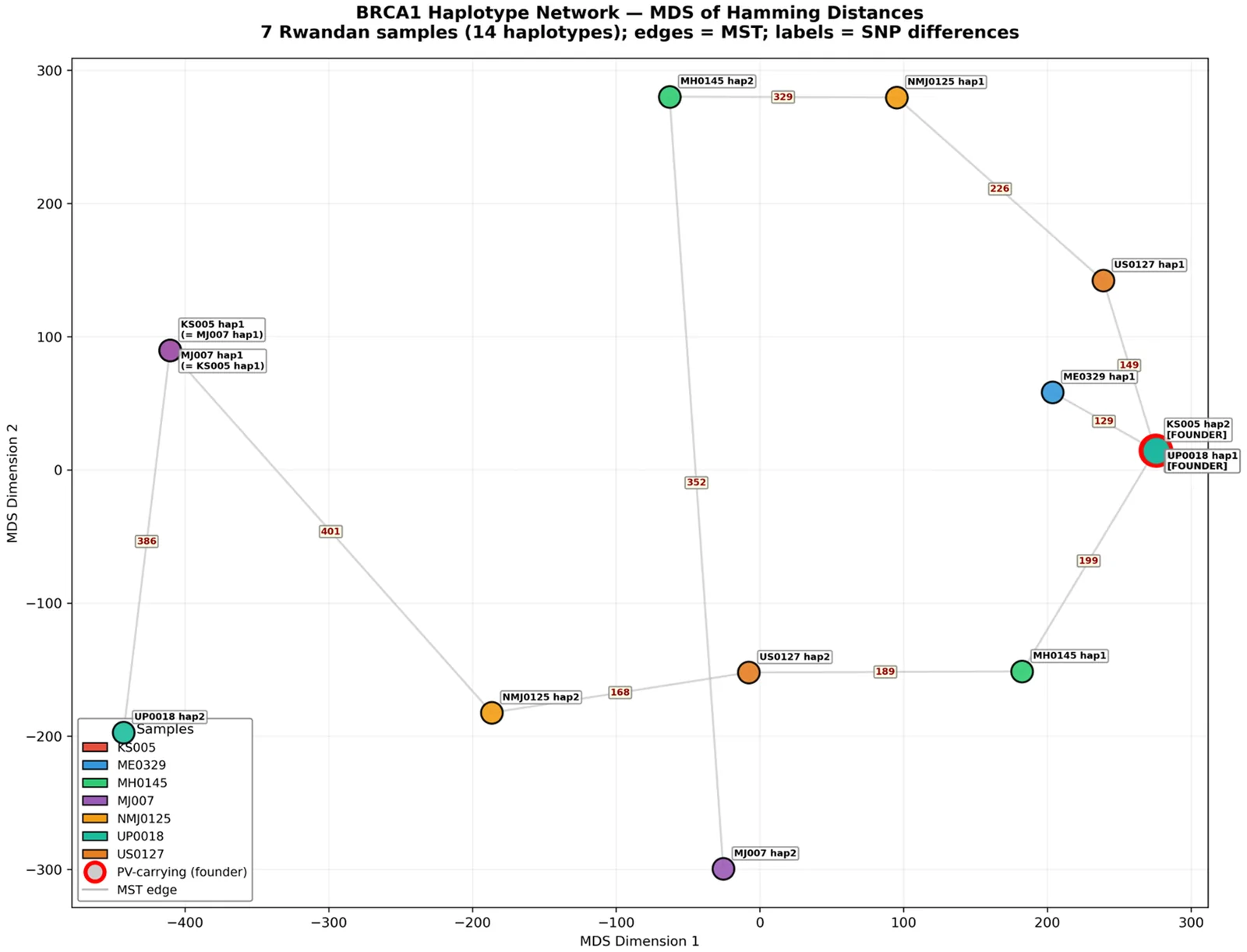
Haplotype network of 14 phased BRCA1 haplotypes.

Population ancestry context from IBS-based MDS of *BRCA1* haplotype sharing in Rwandan vs 1000 Genomes populations is shown in S4 Fig. IBS-based MDS projects the 7 Rwandan samples (black stars with gold edges) onto the haplotype structure of 2,548 individuals from the 1000 Genomes Project at 394 overlapping variant positions Panel A (MDS1 vs MDS2) and Panel B (MDS1 vs MDS3) show that the Rwandan samples are dispersed primarily within and near the African (AFR, red) cluster, consistent with their population of origin. Some Rwandan individuals overlap with AMR and SAS clusters on certain MDS axes, reflecting the limited resolution of 14 tag SNPs for fine-scale population differentiation. The MDS confirms that the *BRCA1* haplotype structure of Rwandan carriers is broadly African but does not closely match any single 1000G sub-population, consistent with the founder haplotype being locally enriched in the Rwandan population.

### Patient relatedness

To formally assess whether the 7 founder PV carriers shared recent common ancestry, we computed KING-robust kinship coefficients and IBS0 fractions across 733,829 autosomal SNPs for all 21 pairwise combinations (C(7,2)). All kinship coefficients fell well below the 0.0442 threshold defining third-degree relatedness (range −0.19 to −0.13; mean −0.17), and IBS0 was approximately 0.07 for every pair — values incompatible with any first-, second-, or third-degree relationship. KING retained all seven individuals as mutually unrelated. These results exclude close or cryptic relatedness and confirm that the shared *BRCA1* haplotype reflects a common founder chromosome rather than recent shared ancestry. KING-robust kinship coefficients and IBS0 fractions for all 21 pairwise combinations of the 7 founder PV carriers are shown in S5 Fig; all pairs fall well below the third-degree relatedness threshold (kinship < 0.0442), confirming that the carriers are genetically unrelated (see also S4 Fig for MDS ancestry context).

### Founder age estimation

We used two approaches to estimate the age of the putative founder PV. First, a simple decay-based estimate was applied. The 7-way shared haplotype block spans chr17:42,650,027–43,231,456 (~581 kb). Using the GRCh38 genetic map, this corresponds to a genetic distance of 68.2375–68.5505 cM, yielding a block length of 0.3129 cM (0.003129 Morgans). Applying the standard decay formula g = 1/(2L), we estimated that the founder arose approximately 160 generations ago, with an estimated age of 4,474 years (assuming 160 generations × 28 yr/gen), or approximately 2,448 BCE, This simple estimate uses the longest contiguous block shared by all 7 carriers and provides a lower bound on the mutation age, since the 7-way block represents the minimum shared region across all haplotypes.

Second, we used a pairwise estimate with bootstrap CI to examine all 21 sample pairs (C(7,2)) using optimal haplotype matching with 2% mismatch tolerance to accommodate phasing errors. The distribution of pairwise shared block lengths was strongly right-skewed. The mean shared block was 0.1424 cM (287.7 kb) (Median shared block: 0.0319 cM; 158.6 kb). Using this approach, we estimated that the PV arose about 351 (95% CI: 242–620) generations ago, with an estimated mean age of ~9,831 years (95% CI: 6,764–17,357 years). The pairwise estimate yields an older age than the simple decay method because it incorporates shorter shared blocks from pairs that share less recent ancestry. Both estimates are consistent with a founder event in the range of ~4,500–10,000 years ago, placing the origin of this *BRCA1* PV in the Neolithic to Bronze Age period. The wide confidence interval (95% CI: 6,764–17,357 years) reflects the limited statistical power of n = 7 carriers. With additional samples, the estimate would narrow considerably. A permutation test comparing the observed 7-way identical-by-state-compatible shared core (~49 kb) against 10,000 random sets of 7 African individuals yielded an empirical p-value of approximately 0.04–0.08 (depending on marker ascertainment threshold), providing suggestive rather than definitive statistical support for founder haplotype sharing. The observed shared core and null distribution are illustrated in S6 Fig.

Taken together, the simple decay estimate (~4,474 years) likely represents a lower bound, while the pairwise mean estimate (~9,831 years) may be inflated by pairs with short, shared blocks due to phasing errors in the small sample. Both estimates are directionally concordant in placing the variant origin well before recent historical times. The data support a broad conclusion that the variant is an ancient, pre-historical founder allele that arose at least several thousand years ago, most plausibly within the range of 4,000–10,000 years; a more precise estimate requires additional carriers.

## Discussion

These results support that c.4065_4068del (p.Asn1355Lysfs*10) represents a founder mutation in Rwanda. Across multiple datasets and populations, *BRCA1* c.4065_4068del (p.Asn1355Lysfs*10) emerges as a clearly pathogenic, recurrent frameshift variant, but current evidence does not yet establish a single, well‑characterized founder origin. The deletion removes four nucleotides in exon 10, producing a frameshift at codon 1355 and a premature stop 10 amino acids downstream, and is consistently classified as pathogenic in ClinVar and clinical knowledgebases. It is very rare in general‑population datasets (only a handful of alleles in gnomAD), yet it has been observed repeatedly in high‑risk breast and ovarian cancer families and unselected cases from Europe, Asia, and the Middle East. In early European series, c.4065_4068del (often referred to as 4184del4) was among the common *BRCA1* PVs in high‑risk breast/ovarian families from northwest England [15], where haplotype analysis suggested that this and another recurrent mutation likely arose on specific ancestral chromosomes shared by multiple families, consistent with founder effects at regional or national scales. Large German and North American cohorts similarly reported this variant among recurrent *BRCA1* PVs, but without definitive evidence for a single pan‑European founder [4,16].

More recent work extends this pattern to Asian and Middle Eastern populations [7,8,18] and again supports recurrence, with hints—but not definitive proof—of region‑specific founder events. In a large Chinese ovarian cancer series (n = 530), c.5470_5477del, c.981_982del, and c.4065_4068del were the three most frequent BRCA1 PVs, strongly suggesting that c.4065_4068del has been established in East Asian populations as a recurrent, and possibly founder, allele [17]. A Pakistani database of *BRCA1/2* variants found c.4065_4068del among 23 recurrent *BRCA1* PVs, and chased its origin alongside other frequent alleles, again pointing to a shared haplotype within that setting [8]. In the Gulf region, both a UAE series and an Emirati hereditary breast/ovarian cancer cohort identified c.4065_4068del as one of the most common *BRCA1* PVs, including four unrelated Emirati carriers in one recent clinic‑based study [7,18,19]. Laitman et al.’s regional synthesis of *BRCA1/2* PVs across Middle Eastern, North African, and South European countries shows that c.4065_4068del appears repeatedly in multiple countries, though it does not reach the very high, multi‑country frequencies of “classical” founder variants such as c.68_69del, c.181T > G, or c.5266dup [9].

Taken together, these reports indicate that *BRCA1* c.4065_4068del is a globally recurrent, clearly deleterious allele that has reached appreciable frequencies in several geographically and ancestrally distinct populations—including northwest Europe [15,20], Germany [4], Pakistan [8], China [17], and the Arabian Gulf [7,18] —rather than being confined to a single, well‑defined founder population. Several lines of evidence suggest that at least some of these clusters are due to local founder effects: (i) enrichment among high‑risk families within specific regions (e.g., northwest England, Pakistan, parts of China), (ii) repeated identification in unrelated Emirati and UAE families, and (iii) the presence of shared extended haplotypes around *BRCA1* 4184del4 in earlier European work [21,22]. However, formal haplotype and coalescent analyses for c.4065_4068del have been limited and region‑specific; no study has yet combined data across continents to infer a single ancestral origin or reconstruct migration paths. The available evidence is compatible with either a relatively old mutation that has spread and drifted in frequency across Eurasia and North Africa, or multiple, independent mutational events at a short deletion‑prone motif.

Our Rwandan observations add a new and important dimension to this literature. Finding c.4065_4068del in 7 of 175 (4%) unselected breast cancer cases—and in 22% of all carriers of PVs—among women without reported close relatedness is striking compared with its rarity in global reference datasets and its generally modest frequencies in other series. Given historical links between East/Central Africa, the Middle East, and the Indian Ocean trading network [18,19,23,24], one plausible hypothesis is that the Rwandan allele shares ancestry with a Middle Eastern or South Asian founder lineage (e.g., Pakistani or Emirati), but this cannot be assumed from frequency data alone. At present, no published study has performed cross‑regional haplotype comparison of African, Middle Eastern, and Asian carriers of c.4065_4068del. Thus, while prior work strongly supports the notion that c.4065_4068del can behave as a founder variant within specific populations (northwest England, parts of Pakistan, China, and the Gulf), the geographic origin of the variant remains unresolved, and its presence in Rwanda could reflect either an independent founder event or introduction via historical gene flow from Middle Eastern or North African sources.

These gaps underscore the need for targeted haplotype and ancestry analyses of c.4065_4068del carriers across diverse populations. In our Rwandan series, high local frequency among apparently unrelated cases strongly suggests a founder effect in this population. By comparing extended haplotypes around *BRCA1* in Rwandan carriers with those reported (or that can be imputed) from European, Middle Eastern, North African, South Asian, and East Asian carriers, future work can test whether the Rwandan founder shares a common ancestral chromosome with any of these groups or represents a distinct African origin. Such analyses would not only clarify the evolutionary history of this clinically important variant but also inform region‑specific genetic testing strategies and cascade screening in Rwanda and neighboring countries.

Our haplotype analyses suggest that the *BRCA1* c.4065_4068del (p.Asn1355Lysfs*10) PVs is an ancient founder allele in the Rwandan population, with an origin most plausibly between roughly 4,000 and 10,000 years ago. Using a simple decay‑based approach applied to the longest haplotype segment shared by all seven carriers (581 kb; 0.3129 cM), we estimated a lower‑bound age of approximately 160 generations, or ~4,474 years (∼2,450 BCE, assuming 28 years per generation). This estimate reflects the minimum time required for recombination to erode a shared ancestral chromosome segment of this length and is constrained by the requirement that the segment be common to all carriers, which biases toward younger, more recent shared ancestry. A complementary pairwise approach that considered all 21 carrier pairs, allowing for a small mismatch tolerance to accommodate phasing error and local haplotype noise, yielded shorter average shared segments (mean 0.1424 cM; median 0.0319 cM) and a correspondingly older inferred age. Under this model, the PV was estimated to have arisen around 351 generations ago (mean ~9,831 years), with a wide 95% confidence interval of 242–620 generations (~6,764–17,357 years). This older estimate reflects the inclusion of pairs who share only short residual haplotypes around *BRCA1*, which may arise from deeper ancestry or from methodological noise in a small sample. Importantly, both methods are directionally concordant in placing the origin of the variant well before recent historical times, in the late Neolithic to Bronze Age period, rather than arising from a recent demographic event such as the last few centuries of migration or admixture.

From a population‑genetic and clinical perspective, these age estimates have several implications. First, they support a true founder effect rather than a de novo or very recent introduction: the variant has persisted through many generations of demographic change, recombination, and potential population bottlenecks, yet remains detectable at appreciable frequency (4% of tested breast cancer cases) in contemporary Rwanda. Second, an origin several thousand years ago is consistent with the possibility that c.4065_4068del may also be present—at low but non‑zero frequency—in neighboring populations that share deep ancestry with Rwandans, even if it has not yet been widely reported in those settings. Third, the lack of a tightly constrained point estimate and the broad confidence interval highlight the limitations of mutation‑age inference from only seven carriers; additional Rwandan and regional carriers, combined with higher‑density phasing and cross‑population haplotype comparison, will be needed to refine both the age estimate and any inference about whether the Rwandan founder shares a common origin with reported carriers in Europe, Asia, or the Middle East.

Overall, the most robust interpretation is that *BRCA1* c.4065_4068del is an old, likely pre‑historical founder variant in this population, with a true age probably between ~4,000 and 10,000 years. This time frame implies that the allele has been segregating through many generations of ancestry and social structure in the Great Lakes region. For clinical genetics and public health, this strengthens the rationale for including c.4065_4068del in targeted or low‑cost testing panels for Rwanda and potentially for neighboring countries, and it motivates further work to map its geographical distribution, refine its ancestral origin, and characterize its penetrance and phenotype spectrum in diverse African settings.

Our previous findings suggest that this variant confers disease aggressiveness based on staging and disease features at presentation, compounded with the triple negative nature of the disease. Elsewhere, this variant has been associated with triple negative disease subtype [11,18], although stage and disease grade were not specified. In our parent study, the variant was not found among patients with prostate cancer.

In conclusion, our analysis reveals that the *BRCA1* c.4065_4068del variant is a pre-historical founder allele that has segregated within the Rwandan population from the late Neolithic or Bronze age, rather than being a result of recent gene flow. Further cross-regional studies are needed to determine if this lineage shares a common ancestor with global clusters or represents a distinct African origin, potentially refining our understanding of hereditary cancer risks across the continent.

## Supporting information

S1 Fig Phased haplotype structure of 7 Rwandan BRCA1 carriers. (DOCX)

S2 Fig Haplotype bifurcation diagram at the BRCA1 pathogenic variant region. (DOCX)

S3 Fig Pairwise shared haplotype block length distribution. (DOCX)

S4 Fig MDS of BRCA1 haplotype sharing: Rwandan vs 1000 Genomes populations. (DOCX)

S5 Fig KING-robust kinship (left) and IBS0 (right) for all 21 pairs of the 7 carriers. (DOCX)

S6 Fig Observed shared core (orange) versus the null distribution from 10,000 random sets of seven 1000G-AFR individuals (blue). Left: 7-way shared core.
(DOCX)

## References

[1] Manirakiza AV, Mutesa L, Mathew CG, Olopade OI. Breast cancer: genetic risk assessment, diagnostics, and therapeutics in African populations. Ann Rev Genomics and Human Genetics. 2025;26. 10.1146/annurev-genom-111522-013953 40294412

[2] Huo D, Hu H, Rhie SK, Gamazon ER, Cherniack AD, Liu J, et al. Comparison of breast cancer molecular features and survival by African and European Ancestry in the cancer genome atlas. JAMA Oncol. 2017;3(12):1654–62. doi: 10.1001/jamaoncol.2017.0595 28472234 PMC5671371

[3] Limenih MA, Mekonnen EG, Birhanu F, Jima BR, Sisay BG, Kassahun EA, et al. Survival patterns among patients with breast cancer in Sub-Saharan Africa: a systematic review and meta-analysis. JAMA Netw Open. 2024;7(5):e2410260. doi: 10.1001/jamanetworkopen.2024.10260 38743426 PMC11094564

[4] German Consortium for Hereditary Breast and Ovarian Cancer. Comprehensive analysis of 989 patients with breast or ovarian cancer provides BRCA1 and BRCA2 mutation profiles and frequencies for the German population. Int J Cancer. 2002;97(4):472–80. 10.1002/ijc.1626 11802209

[5] Kuchenbaecker KB, Hopper JL, Barnes DR, Phillips K-A, Mooij TM, Roos-Blom M-J, et al. Risks of breast, ovarian, and contralateral breast cancer for BRCA1 and BRCA2 mutation carriers. JAMA. 2017;317(23):2402–16. doi: 10.1001/jama.2017.7112 28632866

[6] Park K-S, Lee W, Seong M-W, Kong S-Y, Lee K-A, Ha J-S, et al. A population-based analysis of brca1/2 genes and associated breast and ovarian cancer risk in Korean Patients: A Multicenter Cohort Study. Cancers (Basel). 2021;13(9):2192. doi: 10.3390/cancers13092192 34063308 PMC8125125

[7] Al Ali AAAA, Al Ali MMA, El-Shourbagy DMA-H, Tirmazy SHH, Mirza I, Raza A, et al. Genetic characterization of BRCA1 and BRCA2 variants in cancer and high-risk family screening cohorts in the UAE population. J Cancer Res Clin Oncol. 2025;151(4):146. doi: 10.1007/s00432-025-06188-9 40257527 PMC12011969

[8] Farooq A, Naveed AK, Azeem Z, Ahmad T. Breast and ovarian cancer risk due to prevalence of BRCA1 and BRCA2 variants in Pakistani population: a Pakistani database report. J Oncol. 2011;2011:632870. doi: 10.1155/2011/632870 21559243 PMC3087439

[9] Laitman Y, Friebel TM, Yannoukakos D, Fostira F, Konstantopoulou I, Figlioli G, et al. The spectrum of BRCA1 and BRCA2 pathogenic sequence variants in Middle Eastern, North African, and South European countries. Hum Mutat. 2019;40(11):e1–23. doi: 10.1002/humu.23842 31209999

[10] Adedokun B, Zheng Y, Ndom P, Gakwaya A, Makumbi T, Zhou AY, et al. Prevalence of inherited mutations in breast cancer predisposition genes among women in Uganda and Cameroon. Cancer Epidemiol Biomarkers Prev. 2020;29(2):359–67. doi: 10.1158/1055-9965.EPI-19-0506 31871109 PMC7007381

[11] Rweyemamu LP, Gültaşlar BK, Akan G, Dharsee N, Namkinga LA, Lyantagaye SL, et al. Breast cancer in East Africa: prevalence and spectrum of germline SNV/indel and CNVs in BRCA1 and BRCA2 genes among breast cancer patients in Tanzania. Cancer Medicine. 2023;12(3):3395–409. 10.1002/cam4.5091 PMC9939169 35908255

[12] Ekdahl Hjelm T, Gebremariam TY, Alemu E, Woldegeorgis MA, Borg Å, Fekadu Weldearegay M, et al. Germline pathogenic variants in BRCA1-, BRCA2-, and PALB2-genes among Ethiopian young women and men diagnosed with breast cancer. 2025.

[13] Manirakiza AV, Baichoo S, Uwineza A, Dukundane D, Uwinkindi F, Ngendahayo E, et al. Germline sequence variation in cancer genes in Rwandan breast and prostate cancer cases. NPJ Genom Med. 2024;9(1):61. doi: 10.1038/s41525-024-00446-4 39582020 PMC11586404

[14] Uyisenga JP, Segers K, Lumaka AZ, Mugenzi P, Fasquelle C, Boujemila B, et al. Screening of germline mutations in young Rwandan patients with breast cancers. Mol Genet Genomic Med. 2020;8(11):e1500. doi: 10.1002/mgg3.1500 32959997 PMC7667342

[15] Evans DGR, Neuhausen SL, Bulman M, Young K, Gokhale D, Lalloo F. Haplotype and cancer risk analysis of two common mutations, BRCA1 4184del4 and BRCA2 2157delG, in high risk northwest England breast/ovarian families. J Med Genet. 2004;41(2):e21. doi: 10.1136/jmg.2003.012104 14757871 PMC1735669

[16] Zhang S, Royer R, Li S, McLaughlin JR, Rosen B, Risch HA, et al. Frequencies of BRCA1 and BRCA2 mutations among 1,342 unselected patients with invasive ovarian cancer. Gynecol Oncol. 2011;121(2):353–7. doi: 10.1016/j.ygyno.2011.01.020 21324516

[17] Li J, Han S, Zhang C, Luo Y, Wang L, Wang P, et al. Identification of BRCA1:c.5470_5477del as a founder mutation in Chinese ovarian cancer patients. Front Oncol. 2021;11:655709. doi: 10.3389/fonc.2021.655709 34046351 PMC8148338

[18] Rawashdeh RR, Pederson H, Iskanderian R, Khan F, Altrabulsi B, Ricci S, et al. Germline genetic susceptibility testing among emirati nationals at risk for hereditary breast and ovarian cancer syndrome. JCO Glob Oncol. 2024;10:e2400352. doi: 10.1200/GO-24-00352 39541563

[19] Hurles ME, Sykes BC, Jobling MA, Forster P. The dual origin of the Malagasy in Island Southeast Asia and East Africa: evidence from maternal and paternal lineages. Am J Hum Genet. 2005;76(5):894–901. doi: 10.1086/430051 15793703 PMC1199379

[20] Thomassen M, Hansen TVO, Borg A, Lianee HT, Wikman F, Pedersen IS, et al. BRCA1 and BRCA2 mutations in Danish families with hereditary breast and/or ovarian cancer. Acta Oncol. 2008;47(4):772–7. doi: 10.1080/02841860802004974 18465347

[21] Møller P, Heimdal K, Apold J, Fredriksen A, Borg A, Hovig E, et al. Genetic epidemiology of BRCA1 mutations in Norway. Eur J Cancer. 2001;37(18):2428–34. doi: 10.1016/s0959-8049(01)00299-4 11720839

[22] Scottish/Northern Irish BRCAI/BRCA2 Consortium. BRCA1 and BRCA2 mutations in Scotland and Northern Ireland. Br J Cancer. 2003;88(8):1256–62. doi: 10.1038/sj.bjc.6600840 12698193 PMC2747571

[23] Luis JR, Rowold DJ, Regueiro M, Caeiro B, Cinnioğlu C, Roseman C, et al. The levant versus the horn of africa: evidence for bidirectional corridors of human migrations. Am J Hum Genet. 2004;74(3):532–44. 10.1086/382286 PMC1182266 14973781

[24] Cruciani F, Santolamazza P, Shen P, Macaulay V, Moral P, Olckers A, et al. A back migration from Asia to sub-Saharan Africa is supported by high-resolution analysis of human Y-chromosome haplotypes. Am J Hum Genet. 2002;70(5):1197–214. doi: 10.1086/340257 11910562 PMC447595

