# Supplementary material for: A common pathogenic founder variant in Rwandan breast cancer cases
Source: PLoS Genet. 2026 Nov 2;22(11):e1012180. doi: 10.1371/journal.pgen.1012180 (PMC13739215; doi:10.1371/journal.pgen.1012180)
Supplement: Supplementary file 1 [file pgen.1012180.s007.pdf]

Reviewer Report

Manuscript Title: Founder Haplotype Characterization and Age Estimation of a Pathogenic BRCA1 Variant in Rwandan Breast Cancer Patients

This manuscript addresses a scientifically important question and presents potentially novel findings regarding hereditary breast cancer genetics in Rwanda. However, substantial revisions are required to strengthen the evidence supporting a founder effect, clarify methodological assumptions, improve the interpretation of founder age estimates, and more thoroughly discuss the limitations inherent to the small sample size and population-genetic inference framework. With these revisions, the study could make a valuable contribution to the field of cancer genetics and African genomics.

Major Comments

1- The most significant limitation of the study is the inclusion of only seven unrelated mutation carriers. Although the rarity of the variant may justify the limited sample size, the small number of carriers substantially affects accuracy of haplotype reconstruction, age estimation… The authors partially acknowledge this challenge by allowing a 2% mismatch rate when identifying shared haplotype blocks. However, this issue deserves a much more comprehensive discussion.

2- The presence of a shared haplotype among carriers supports, but does not conclusively prove, a founder effect. Several important pieces of evidence are missing, frequency of the variant in broader African datasets; presence or absence of the variant in neighboring populations.

3- Several analyses, including EHH and bifurcation analyses, rely on a nearby proxy SNP located 521 bp from the pathogenic variant. While this approach is understandable because the causal variant was dropped during imputation, the manuscript does not provide sufficient evidence that the proxy accurately captures the pathogenic allele.

4- There is a lack of formal statistical testing to evaluate whether the observed haplotype sharing exceeds expectations by chance. Population comparisons rely on only 14 overlapping tag SNPs, providing limited resolution for fine-scale population structure analyses.

5- The claim that the founder haplotype may be private to or enriched in Rwanda/East Africa should be softened. The absence of a matching haplotype in the 1000 Genomes dataset does not necessarily imply geographic specificity, particularly because Rwandan populations are not specifically represented and the comparative marker density is low.

6- The founder age estimates represent the most uncertain aspect of the study. The large discrepancy between the simple decay estimate (~4,500 years) and the pairwise estimate (~9,800 years) indicates considerable sensitivity to methodological assumptions and limited precision. Consequently, the data support only a broad estimate that the variant likely arose several thousand years ago.

7- Although the carriers were not known to be related, no formal genomic relatedness analyses (e.g., KING, IBD, or kinship coefficients) were presented. Therefore, distant cryptic relatedness cannot be excluded.

Minor Comments

1- I recommend adopting ACMG-compliant terminology throughout the manuscript and consistently using “pathogenic variant (PV)” instead of alternating between, mutation, pathogenic mutation, frameshift mutation.

2- The result section should highlight the study finding rather than listing and commenting the figures and tables provided

3- In the result section, tables 1 and 2 status should be clarified (study results or supporting information)
