# Supplementary material for: A common pathogenic founder variant in Rwandan breast cancer cases
Source: PLoS Genet. 2026 Nov 2;22(11):e1012180. doi: 10.1371/journal.pgen.1012180 (PMC13739215; doi:10.1371/journal.pgen.1012180)
Supplement: Supplementary file 2 [file pgen.1012180.s001.docx]

**Supplementary Figure 1. Phased haplotype structure of 7 Rwandan BRCA1 carriers.**

**Supplementary Figure 2. Haplotype bifurcation diagram at the BRCA1 pathogenic variant region.**

**Supplementary Figure 3. Pairwise shared haplotype block length distribution.**

**Supplementary Figure 4. MDS of BRCA1 haplotype sharing: Rwandan vs 1000 Genomes populations.**
