# Supplementary material for: A common pathogenic founder variant in Rwandan breast cancer cases
Source: PLoS Genet. 2026 Nov 2;22(11):e1012180. doi: 10.1371/journal.pgen.1012180 (PMC13739215; doi:10.1371/journal.pgen.1012180)
Supplement: Supplementary file 3 [file pgen.1012180.s002.docx]

**Supplementary Figure S2. Haplotype bifurcation diagram at the BRCA1 pathogenic variant region.**
