# Supplementary material for: A common pathogenic founder variant in Rwandan breast cancer cases
Source: PLoS Genet. 2026 Nov 2;22(11):e1012180. doi: 10.1371/journal.pgen.1012180 (PMC13739215; doi:10.1371/journal.pgen.1012180)
Supplement: Supplementary file 6 [file pgen.1012180.s005.docx]

**Supplementary Figure S5. KING-robust kinship (left) and IBS0 (right) for all 21 pairs of the 7 carriers.**
