# Supplementary material for: A common pathogenic founder variant in Rwandan breast cancer cases
Source: PLoS Genet. 2026 Nov 2;22(11):e1012180. doi: 10.1371/journal.pgen.1012180 (PMC13739215; doi:10.1371/journal.pgen.1012180)
Supplement: Supplementary file 7 [file pgen.1012180.s006.docx]

**Supplementary Figure S6. Observed shared core (orange) versus the null distribution from 10,000 random sets of seven 1000G-AFR individuals (blue). Left: 7-way shared core.**
