# Supplementary material for: A common pathogenic founder variant in Rwandan breast cancer cases
Source: PLoS Genet. 2026 Nov 2;22(11):e1012180. doi: 10.1371/journal.pgen.1012180 (PMC13739215; doi:10.1371/journal.pgen.1012180)
Supplement: Supplementary file 8 [file pgen.1012180.s009.docx]

We thank the reviewers for the constructive feedback on our manuscript. We have addressed all the comments and provided context as follows:

**Reviewer comment**

*R1: “The authors should use data from continental African samples to better understand the history of this putative founder mutation. For example, these references samples are available through H3Africa.*

*R2: “Several important pieces of evidence are missing: frequency of the variant in broader African datasets; presence or absence of the variant in neighboring populations.”*

**Response**

As suggested, we examined a continental-African reference cohort — whole-exome data from 1,561 individuals in the MADCaP consortium (896 cases, 665 controls). The pathogenic frameshift BRCA1 c.4065_4068del (chr17:43,091,462 CTTGA>C) was present at very low frequency: a single heterozygous carrier among 1,559 genotyped individuals (carrier frequency 0.06%; allele frequency 3.2×10⁻⁴). Thus the variant is not confined to Rwanda, but is roughly 60-fold rarer in the broader African population than among our Rwandan breast-cancer cases (4%), consistent with a founder allele that has reached appreciable local frequency in Rwanda while remaining exceedingly rare across the continent. This African- specific estimate complements the gnomAD data, which under-represent African and, in particular, Rwandan populations.

**Addition in Text: See Results Section, Page 7-8**

*R1: “… in order to claim a founder mutation, this definitely needs genetic confirmation. However, the analysis entitled relatedness does not actually test for that. Instead, it is an MDS analysis… With WES data… degree of relatedness can be estimated.” R2: “…no formal genomic relatedness analyses (e.g., KING, IBD, or kinship coefficients) were presented. Therefore, distant cryptic relatedness cannot be excluded”*

**Response**

To formally test relatedness rather than population structure, we computed KING-robust kinship coefficients and IBS0 across 733,829 autosomal SNPs from the whole-exome data for all 21 pairs of carriers. All pairwise kinship coefficients were well below the 0.0442 threshold that defines third-degree relatedness (range −0.19 to −0.13; mean −0.17), and IBS0 was ≈ 0.07 for every pair — values incompatible with any first-, second- or third-degree relationship (KING retained all seven individuals as mutually unrelated). These results exclude close or cryptic relatedness and confirm that the shared haplotype surrounding BRCA1 c.4065_4068del reflects a common founder chromosome rather than recent shared ancestry. We have replaced the previous MDS-based ‘relatedness’ analysis, which described population structure, with this formal kinship analysis,

**Addition in Text: See “Methods” Section, Page 6; under “Results” section, page 10 Figure added as Supplementary Figure 5 (***KING-robust kinship (left) and IBS0 (right) for all 21 pairs of the 7 carriers)*

**Reviewers’ comments:**

*“I don’t understand why phasing wasn’t done for all variants in the gene. The authors restricted this to common variants with MAF > 5%. Also, they only selected 48 tag SNPs.” R2: “…increase the tag SNPs to not exclude the rare variants… Population comparisons rely on only 14 overlapping tag SNPs, providing limited resolution”*

**Response**

We thank the reviewers and have clarified the Methods. With only seven carriers a within-sample MAF ≥ 5% threshold is nearly inert — any variant seen even once among 14 chromosomes already exceeds 5% — so removing it changed the biallelic-SNP count only marginally (1,035 → 1,114). The variants genuinely excluded were low-call-rate sites and, importantly, indels, including the pathogenic frameshift, which is why a proxy SNP was previously required. We have rebuilt the marker panel from all biallelic variants (SNPs and indels) with no MAF filter (533 markers, including the pathogenic variant), and for the population comparison now use all shared-block variants overlapping the reference panels rather than 48 LD-pruned tag SNPs; this increases the markers overlapping 1000 Genomes AFR from 14 to 394 and adds 112 overlapping markers in the MADCaP African cohort, substantially improving resolution.

Reviewers’ comments:

*R2: “EHH and bifurcation analyses rely on a nearby proxy SNP located 521 bp from the pathogenic variant… the manuscript does not provide sufficient evidence that the proxy accurately captures the pathogenic allele”*

Response: The pathogenic frameshift is directly genotyped in all seven carriers (each heterozygous); it was excluded only during reference-panel phasing, because indels absent from the panel are dropped — hence our earlier use of a proxy SNP. We have now re-phased the region so that the pathogenic variant itself is retained and used as the focal marker, removing reliance on a proxy. For completeness we characterised the proxy quantitatively: it lies on the pathogenic haplotype in six of the seven carriers, with linkage disequilibrium D' = 0.67 and r² = 0.33 in the phased data (the single discordant carrier is most likely a statistical-phasing switch error at this sample size; assuming so raises the estimate to D' = 1.0, r² = 0.75). Because the proxy allele is common in African populations (≈0.18–0.22), it also occurs on non-carrier haplotypes and over-represents carriers, so it is an imperfect tag. We further note that extended-haplotype-homozygosity and bifurcation statistics are sensitive to statistical-phasing switch errors at this sample size, and we have accordingly de-emphasized EHH magnitudes in favor of the genotype-based shared haplotype block and the formal relatedness analysis as the primary evidence for a common founder chromosome.

**Addition in text: See under “Methods” section, page 5, and under “Results” page 8. Figure 4a replaced in the Figures files.**

*Reviewer’s comment: “There is a lack of formal statistical testing to evaluate whether the observed haplotype sharing exceeds expectations by chance.”*

Response: To formally test whether the haplotype sharing exceeds chance, we performed a permutation test. Using markers ascertained independently from 1000 Genomes AFR (MAF ≥ 0.10) to avoid ascertainment bias, we measured the region around the pathogenic variant over which all seven carriers remain mutually identical-by-state compatible (no opposite-homozygous site) and compared it with 10,000 random sets of seven African individuals. The observed shared core (~49 kb) was larger than expected by chance, with modest statistical support (empirical p ≈ 0.04–0.08 depending on the marker-ascertainment threshold and on how sites without a called variant are treated; p ≈ 0.07 without that coverage assumption); we therefore describe this as suggestive rather than definitive evidence. We also note that this strict, tolerance-free core is considerably shorter than the 581 kb block reported with a 2% mismatch tolerance; we now present the ~49 kb identical core as the shared segment and describe the 581 kb figure as a tolerance-based outer bound. A short-shared core is what is expected for an ancient founder allele whose flanking haplotype has been eroded by recombination, consistent with our age estimates.

**Addition in text: See under “Results” section, page 11; Added Supplementary Figure S6**

Reviewers’ comments: *R1: “The age of the founder event cannot be reliably estimated – the two methods give very different results… be explicit about the findings which have sufficient confidence.” R2: “The founder age estimates represent the most uncertain aspect of the study… the data support only a broad estimate that the variant likely arose several thousand years ago.”*

Response: We agree that the age estimate is the most uncertain aspect of the study and have substantially revised its presentation. We clarify that our two original figures were not two independent methods but the same decay formula applied to two different shared-block lengths; the estimate is therefore dominated by how the block is defined and how much mismatch tolerance is permitted. We now show that the strict, tolerance-free shared core is much shorter than the 581 kb block, so the ~4,500-year value is best interpreted as a lower bound, and that alternative reasonable assumptions yield considerably older ages with very wide confidence intervals. Accordingly, we no longer report a single precise age; we state only the robustly supported conclusion — that the variant is an ancient, pre-historical founder allele that arose at least several thousand years ago — and we explicitly attribute the residual uncertainty to the small number of carriers.

Addition in text: See under “Results” section, page 11; Additional Supplementary Figure S7 added.

Minor Comments:

Reviewer’s comment: *“I recommend adopting ACMG-compliant terminology throughout the manuscript and consistently using “pathogenic variant (PV)” instead of alternating between, mutation, pathogenic mutation, frameshift mutation.*”

Response: This has been addressed throughout the manuscript text

Reviewer’s comments: “*The result section should highlight the study finding rather than listing and commenting the figures and tables provided”*

Response: Addressed under “Results” section.

3- In the result section, tables 1 and 2 status should be clarified (study results or supporting information)

Response: Addressed under “Results” section.
