# Supplementary material for: A common pathogenic founder variant in Rwandan breast cancer cases
Source: PLoS Genet. 2026 Nov 2;22(11):e1012180. doi: 10.1371/journal.pgen.1012180 (PMC13739215; doi:10.1371/journal.pgen.1012180)
Supplement: Peer review history

# Peer review: Decision Letter 0

**DOI:** 10.1371/journal.pgen.1012180.r001

**Authors:** Kent Hunter, Heather Cordell

8 Jul 2026

PGENETICS-D-26-00537

A Common Pathogenic Founder Variant in Rwandan Breast Cancer Cases

PLOS Genetics

Dear Dr. Manirakiza,

Thank you for submitting your manuscript to PLOS Genetics. After careful consideration, we feel that it has merit but does not fully meet PLOS Genetics's publication criteria as it currently stands. Therefore, we invite you to submit a revised version of the manuscript that addresses the points raised during the review process.

Please submit your revised manuscript by Sep 06 2026 11:59PM. If you will need more time than this to complete your revisions, please reply to this message or contact the journal office at plosgenetics@plos.org. When you're ready to submit your revision, log on to https://www.editorialmanager.com/pgenetics/ and select the 'Submissions Needing Revision' folder to locate your manuscript file.

Please include the following items when submitting your revised manuscript:

* A letter that responds to each point raised by the editor and reviewer(s). You should upload this letter as a separate file labeled 'Response to Reviewers'. This file does not need to include responses to any formatting updates and technical items listed in the 'Journal Requirements' section below.

* A marked-up copy of your manuscript that highlights changes made to the original version. You should upload this as a separate file labeled 'Revised Manuscript with Track Changes'.

* An unmarked version of your revised paper without tracked changes. You should upload this as a separate file labeled 'Manuscript'.

If you would like to make changes to your financial disclosure, competing interests statement, or data availability statement, please make these updates within the submission form at the time of resubmission. Guidelines for resubmitting your figure files are available below the reviewer comments at the end of this letter.

As the corresponding author, your ORCID iD is verified in the submission system and will appear in the published article. PLOS supports the use of ORCID, and we encourage all coauthors to register for an ORCID iD and use it as well. Please encourage your coauthors to verify their ORCID iD within the submission system before final acceptance, as unverified ORCID iDs will not appear in the published article. Only the individual author can complete the verification step; PLOS staff cannot verify ORCID iDs on behalf of authors.

We look forward to receiving your revised manuscript.

Kind regards,

Heather J Cordell

Academic Editor

PLOS Genetics

Kent Hunter

Section Editor

PLOS Genetics

Aimée Dudley

Editor-in-Chief

PLOS Genetics

Anne Goriely

Editor-in-Chief

PLOS Genetics

**Additional Editor Comments (if provided):**

**Journal Requirements:**

If the reviewer comments include a recommendation to cite specific previously published works, please review and evaluate these publications to determine whether they are relevant and should be cited. There is no requirement to cite these works unless the editor has indicated otherwise.

1) Please ensure that the CRediT author contributions listed for every co-author are completed accurately and in full.

At this stage, the following Authors/Authors require contributions: Achille VC. Manirakiza, Shakuntala Baichoo, Annette Uwineza, Damas Dukundane, Eulade Rugengamanzi, Janviere Mutamuliza, Allison Niragira, Raissa Muvunyi, Jeffrey Besada, Sarah Nielsen, Brianna Bucknor, Diane R Koeller, Caroline Andrews, Leon Mutesa, Temidayo Fadelu, and Timothy R Rebbeck. Please ensure that the full contributions of each author are acknowledged in the "Add/Edit/Remove Authors" section of our submission form.

The list of CRediT author contributions may be found here: https://journals.plos.org/plosgenetics/s/authorship#loc-author-contributions

2) Please provide an Author Summary. This should appear in your manuscript between the Abstract (if applicable) and the Introduction, and should be 150-200 words long. The aim should be to make your findings accessible to a wide audience that includes both scientists and non-scientists. Sample summaries can be found on our website under Submission Guidelines:

https://journals.plos.org/plosgenetics/s/submission-guidelines#loc-parts-of-a-submission

3) We do not publish any copyright or trademark symbols that usually accompany proprietary names, eg ©,  ®, or TM  (e.g. next to drug or reagent names). Therefore please remove all instances of trademark/copyright symbols throughout the text, including:

- ® on page: 4.

4) Your manuscript is missing the following sections: Introduction.  Please ensure all required sections are present and in the correct order. Make sure section heading levels are clearly indicated in the manuscript text, and limit sub-sections to 3 heading levels. An outline of the required sections can be consulted in our submission guidelines here:

https://journals.plos.org/plosgenetics/s/submission-guidelines#loc-parts-of-a-submission

5) Please upload all main figures as separate Figure files in .tif or .eps format. For more information about how to convert and format your figure files please see our guidelines:

https://journals.plos.org/plosgenetics/s/figures

6) Please amend your detailed Financial Disclosure statement. This is published with the article. It must therefore be completed in full sentences and contain the exact wording you wish to be published.

i) State the initials, alongside each funding source, of each author to receive each grant. For example: "This work was supported by the National Institutes of Health (####### to AM; ###### to CJ) and the National Science Foundation (###### to AM)."

ii) State what role the funders took in the study. If the funders had no role in your study, please state: "The funders had no role in study design, data collection and analysis, decision to publish, or preparation of the manuscript."

iii) If any authors received a salary from any of your funders, please state which authors and which funders..

If you did not receive any funding for this study, please simply state: u201cThe authors received no specific funding for this work.u201d

7)We have amended your Competing Interest statement to comply with journal style. We kindly ask that you double check the statement and let us know if anything is incorrect.

**Reviewers' comments:**

Reviewer's Responses to Questions

**Comments to the Authors:**

**Please note here if the review is uploaded as an attachment.**

Reviewer #1: The present paper by Manirakiza et al applies population genetics methods to genotype data from seven Rwandan participants with a suspected BRCA1 founder mutation. The mutation was identified in a previous publication by the authors.

Overall, there is a strong rationale for investigating whether the reported frameshift mutation is a founder mutation. However, the small sample size severely limit the ability to draw some of the conclusions. Whilst the authors in all likelihood cannot identify more participants, the choice of methods, choice of reference samples and reporting of results all require major improvments.

Comments

Reference samples: The authors use the 1000G data as a reference and for some of the analyses that is acceptable as it enables a global view. However, we already know that this particular mutation is virtually absent in Gnomad so there is not that much comparisons with 1000G can contribute. Instead, the authors should use data from continental African samples to better understand the history of this putative founder mutation. For example, these reference samples are available through H3Africa.

Relatedness: The samples are reported to be unrelated and in order to claim a founder mutation, this definitely needs genetic confirmation. However, the analysis entitled relatedness does not actually test for that. Instead it is an MDS analyses with 1000G that can tell about population structure. With WES data for these samples, degree of relatedness can be estimated.

Context: No context or local knowledge are offered but these would be vital to interpret the findings. Are the individuals from the same ethic subgroup within Rwanda or different ones? Are they from the same geographic region within the country? Is this an ethic group present in other coutrnies ?Could the mutation be found elsewhere? Have similar analyses been done in other samples from the country or neighbouring country and was the same mutation found or not?

Methods: I don’t understand why phasing wasn’t done for all variants in the gene. The authors restricted this to common variants with MAF > 5%. Also they only selected 48 tag SNPs. Is the haplotype present in any other samples from H3A or other references? Phasing is difficult for such a small sample size. Are there any other samples from the same region available that the data could be combined with?

Value of the work: The age of the founder event cannot be reliably estimated – the two methods give very different results. We already knew that the mutation is not present in other groups from Gnomad, so I am not entirely sure what the analyses really reveal. It would be helpful if the authors could be explicit about the findings which have sufficient confidence and how they add to prior knowledge.

The abstract needs improvement as vital information is missing that makes it hard to interperet the findings and understand the incremental value of this work. It is not clear that these are individuals with early onset BC or family history of BC and that they are all triple negative. Nor that these are published results.

Language around the founder mutations and haplotype should be improved, e.g. “Rwandan founder haplotype” –This could be stigmatising. Better label by mutation of interest. We don’t know if founder mutation is found beyond Rwanda or just within one ethnic subgroup within Rwanda.

Results are not well described. It reads like a list of Figure/table legends. There are many whilst the description of key results is often limited.

Reviewer #2: Reviewer Report

Manuscript Title: Founder Haplotype Characterization and Age Estimation of a Pathogenic BRCA1 Variant in Rwandan Breast Cancer Patients

This manuscript addresses a scientifically important question and presents potentially novel findings regarding hereditary breast cancer genetics in Rwanda. However, substantial revisions are required to strengthen the evidence supporting a founder effect, clarify methodological assumptions, improve the interpretation of founder age estimates, and more thoroughly discuss the limitations inherent to the small sample size and population-genetic inference framework. With these revisions, the study could make a valuable contribution to the field of cancer genetics and African genomics.

Major Comments

1- The most significant limitation of the study is the inclusion of only seven unrelated mutation carriers. Although the rarity of the variant may justify the limited sample size, the small number of carriers substantially affects accuracy of haplotype reconstruction, age estimation… The authors partially acknowledge this challenge by allowing a 2% mismatch rate when identifying shared haplotype blocks. However, this issue deserves a much more comprehensive discussion.

2- The presence of a shared haplotype among carriers supports, but does not conclusively prove, a founder effect. Several important pieces of evidence are missing, frequency of the variant in broader African datasets; presence or absence of the variant in neighboring populations.

3- Several analyses, including EHH and bifurcation analyses, rely on a nearby proxy SNP located 521 bp from the pathogenic variant. While this approach is understandable because the causal variant was dropped during imputation, the manuscript does not provide sufficient evidence that the proxy accurately captures the pathogenic allele.

4- There is a lack of formal statistical testing to evaluate whether the observed haplotype sharing exceeds expectations by chance. Population comparisons rely on only 14 overlapping tag SNPs, providing limited resolution for fine-scale population structure analyses.

5- The claim that the founder haplotype may be private to or enriched in Rwanda/East Africa should be softened. The absence of a matching haplotype in the 1000 Genomes dataset does not necessarily imply geographic specificity, particularly because Rwandan populations are not specifically represented and the comparative marker density is low.

6- The founder age estimates represent the most uncertain aspect of the study. The large discrepancy between the simple decay estimate (~4,500 years) and the pairwise estimate (~9,800 years) indicates considerable sensitivity to methodological assumptions and limited precision. Consequently, the data support only a broad estimate that the variant likely arose several thousand years ago.

7- Although the carriers were not known to be related, no formal genomic relatedness analyses (e.g., KING, IBD, or kinship coefficients) were presented. Therefore, distant cryptic relatedness cannot be excluded.

Minor Comments

1- I recommend adopting ACMG-compliant terminology throughout the manuscript and consistently using “pathogenic variant (PV)” instead of alternating between, mutation, pathogenic mutation, frameshift mutation.

2- The result section should highlight the study finding rather than listing and commenting the figures and tables provided

3- In the result section, tables 1 and 2 status should be clarified (study results or supporting information)

**********

**Have all data underlying the figures and results presented in the manuscript been provided?**

Large-scale datasets should be made available via a public repository as described in the *PLOS Genetics*  data availability policy, and numerical data that underlies graphs or summary statistics should be provided in spreadsheet form as supporting information.

Reviewer #1: None

Reviewer #2: Yes

**********

PLOS authors have the option to publish the peer review history of their article (what does this mean?). If published, this will include your full peer review and any attached files.

If you choose “no”, your identity will remain anonymous but your review may still be made public.

**Do you want your identity to be public for this peer review?**  For information about this choice, including consent withdrawal, please see our Privacy Policy.

Reviewer #1: No

Reviewer #2: No

[NOTE: If reviewer comments were submitted as an attachment file, they will be attached to this email and accessible via the submission site. Please log into your account, locate the manuscript record, and check for the action link "View Attachments". If this link does not appear, there are no attachment files.]

**Figure resubmission:**

While revising your submission, we strongly recommend that you use PLOS’s NAAS tool (https://ngplosjournals.pagemajik.ai/artanalysis) to test your figure files. NAAS can convert your figure files to the TIFF file type and meet basic requirements (such as print size, resolution), or provide you with a report on issues that do not meet our requirements and that NAAS cannot fix.

After uploading your figures to PLOS’s NAAS tool - https://ngplosjournals.pagemajik.ai/artanalysis, NAAS will process the files provided and display the results in the "Uploaded Files" section of the page as the processing is complete. If the uploaded figures meet our requirements (or NAAS is able to fix the files to meet our requirements), the figure will be marked as "fixed" above. If NAAS is unable to fix the files, a red "failed" label will appear above. When NAAS has confirmed that the figure files meet our requirements, please download the file via the download option, and include these NAAS processed figure files when submitting your revised manuscript.

**Reproducibility:**

To enhance the reproducibility of your results, we recommend that authors of applicable studies deposit laboratory protocols in protocols.io, where a protocol can be assigned its own identifier (DOI) such that it can be cited independently in the future. Additionally, PLOS ONE offers an option to publish peer-reviewed clinical study protocols. Read more information on sharing protocols at https://plos.org/protocols?utm_medium=editorial-email&utm_source=authorletters&utm_campaign=protocols

Attachment Submitted filename: Reviewer Report_BRCA1 Rwanda.pdf

# Author comment: Author response to Decision Letter 1

**DOI:** 10.1371/journal.pgen.1012180.r002

7 Aug 2026

Attachment Submitted filename: Response to Reviewers.docx

# Peer review: Decision Letter 1

**DOI:** 10.1371/journal.pgen.1012180.r003

**Authors:** Heather Cordell, Hongbin Ji

13 Oct 2026

Dear Dr Manirakiza,

We are pleased to inform you that your manuscript entitled "A Common Pathogenic Founder Variant in Rwandan Breast Cancer Cases" has been editorially accepted for publication in PLOS Genetics. Congratulations!

Before your submission can be formally accepted and sent to production you will need to complete our formatting changes, which you will receive in a follow up email. Please be aware that it may take several days for you to receive this email; during this time no action is required by you. Please note: the accept date on your published article will reflect the date of this provisional acceptance, but your manuscript will not be scheduled for publication until the required changes have been made.

Once your paper is formally accepted, an uncorrected proof of your manuscript will be published online ahead of the final version, unless you’ve already opted out via the online submission form. If, for any reason, you do not want an earlier version of your manuscript published online or are unsure if you have already indicated as such, please let the journal staff know immediately at plosgenetics@plos.org.

In the meantime, please log into Editorial Manager at https://www.editorialmanager.com/pgenetics/, click the "Update My Information" link at the top of the page, and update your user information to ensure an efficient production and billing process. Note that PLOS requires an ORCID iD for all corresponding authors. Therefore, please ensure that you have an ORCID iD and that it is validated in Editorial Manager. To do this, go to ‘Update my Information’ (in the upper left-hand corner of the main menu), and click on the Fetch/Validate link next to the ORCID field.  This will take you to the ORCID site and allow you to create a new iD or authenticate a pre-existing iD in Editorial Manager.

If you have a press-related query, or would like to know about making your underlying data available (as you will be aware, this is required for publication), please see the end of this email. If your institution or institutions have a press office, please notify them about your upcoming article at this point, to enable them to help maximise its impact. Inform journal staff as soon as possible if you are preparing a press release for your article and need a publication date.

Thank you again for supporting open-access publishing; we are looking forward to publishing your work in PLOS Genetics!

Yours sincerely,

Heather J Cordell

Academic Editor

PLOS Genetics

Hongbin Ji

Section Editor

PLOS Genetics

Aimée Dudley

Editor-in-Chief

PLOS Genetics

Anne Goriely

Editor-in-Chief

PLOS Genetics

www.plosgenetics.org

BlueSky: @plos.bsky.social

----------------------------------------------------

Comments from the reviewers (if applicable):

Reviewer's Responses to Questions

**Comments to the Authors:**

**Please note here if the review is uploaded as an attachment.**

Reviewer #2: All comments have been adressed in the revised document.

**********

**Have all data underlying the figures and results presented in the manuscript been provided?**

Large-scale datasets should be made available via a public repository as described in the *PLOS Genetics*  data availability policy, and numerical data that underlies graphs or summary statistics should be provided in spreadsheet form as supporting information.

Reviewer #2: Yes

**********

PLOS authors have the option to publish the peer review history of their article (what does this mean?). If published, this will include your full peer review and any attached files.

If you choose “no”, your identity will remain anonymous but your review may still be made public.

**Do you want your identity to be public for this peer review?**  For information about this choice, including consent withdrawal, please see our Privacy Policy.

Reviewer #2: No

----------------------------------------------------

**Data Deposition**

If you have submitted a Research Article or Front Matter that has associated data that are not suitable for deposition in a subject-specific public repository (such as GenBank or ArrayExpress), one way to make that data available is to deposit it in the Dryad Digital Repository. As you may recall, we ask all authors to agree to make data available; this is one way to achieve that. A full list of recommended repositories can be found on our website.

The following link will take you to the Dryad record for your article, so you won't have to re‐enter its bibliographic information, and can upload your files directly:

http://datadryad.org/submit?journalID=pgenetics&manu=PGENETICS-D-26-00537R1

More information about depositing data in Dryad is available at http://www.datadryad.org/depositing. If you experience any difficulties in submitting your data, please contact help@datadryad.org for support.

Additionally, please be aware that our data availability policy requires that all numerical data underlying display items are included with the submission, and you will need to provide this before we can formally accept your manuscript, if not already present.

----------------------------------------------------

**Press Queries**

If you or your institution will be preparing press materials for this manuscript, or if you need to know your paper's publication date for media purposes, please inform the journal staff as soon as possible so that your submission can be scheduled accordingly. Your manuscript will remain under a strict press embargo until the publication date and time. This means an early version of your manuscript will not be published ahead of your final version. PLOS Genetics may also choose to issue a press release for your article. If there's anything the journal should know or you'd like more information, please get in touch via plosgenetics@plos.org.

# Editor report: Acceptance letter

**DOI:** 10.1371/journal.pgen.1012180.r004

**Authors:** Heather Cordell, Hongbin Ji

PGENETICS-D-26-00537R1

A Common Pathogenic Founder Variant in Rwandan Breast Cancer Cases

Dear Dr Manirakiza,

We are pleased to inform you that your manuscript entitled "A Common Pathogenic Founder Variant in Rwandan Breast Cancer Cases" has been formally accepted for publication in PLOS Genetics! Your manuscript is now with our production department and you will be notified of the publication date in due course.

The corresponding author will soon be receiving a typeset proof for review, to ensure errors have not been introduced during production. Please review the PDF proof of your manuscript carefully, as this is the last chance to correct any errors. Please note that major changes, or those which affect the scientific understanding of the work, will likely cause delays to the publication date of your manuscript.

Soon after your final files are uploaded, unless you have opted out or your manuscript is a front-matter piece, the early version of your manuscript will be published online. The date of the early version will be your article's publication date. The final article will be published to the same URL, and all versions of the paper will be accessible to readers.

For Research Articles, you will receive an invoice from PLOS for your publication fee after your manuscript has reached the completed accept phase. If you receive an email requesting payment before acceptance or for any other service, this may be a phishing scheme. Learn how to identify phishing emails and protect your accounts at https://explore.plos.org/phishing.

Thank you again for supporting PLOS Genetics and open-access publishing. We are looking forward to publishing your work!

With kind regards,

Sharmila Kamatchi

PLOS Genetics

On behalf of:

The PLOS Genetics Team

Carlyle House, Carlyle Road, Cambridge CB4 3DN | United Kingdom

plosgenetics@plos.org | +44 (0) 1223-442823

plosgenetics.org | Twitter: @PLOSGenetics
